# The Impact of Physical Activity on Metabolic Health and Cognitive Function in Postmenopausal Women: A Cross-Sectional Study

**DOI:** 10.3390/metabo15070420

**Published:** 2025-06-20

**Authors:** Kinga Mruczyk, Rafał W. Wójciak, Marta Molska, Ewa Śliwicka, Tomasz Podgórski, Aleksandra Skoczek-Rubińska, Anna Borowiecka, Angelika Cisek-Woźniak

**Affiliations:** 1 Department of Dietetics, Faculty of Physical Culture in Gorzów Wlkp., Poznan University of Physical Education, 61-871 Poznań, Poland; m.molska@awf-gorzow.edu.pl (M.M.); a.cisek@awf-gorzow.edu.pl (A.C.-W.); 2 Department of Clinical Psychology, University of Medical Sciences, 61-701 Poznań, Poland; rafwoj@ump.edu.pl; 3 Department of Biochemistry, Poznan University of Physical Education, 61-871 Poznań, Poland; sliwicka@awf.poznan.pl (E.Ś.); podgorski@awf.poznan.pl (T.P.); 4 Clinic REHA-MED., 66-400 Gorzów Wielkopolski, Poland

**Keywords:** exercise, BDNF, brain-derived neurotrophic factor, cognition, metabolic status, menopause

## Abstract

**Background**: This study aimed to evaluate the impact of physical activity levels on selected biochemical markers (glucose, insulin, cholesterol, triglycerides, interleukin-6 [IL-6]), brain-derived neurotrophic factor (BDNF), cognitive functions, and additional macronutrient intake in postmenopausal women. **Method**: A total of 72 generally healthy women aged 55–73 from western Poland participated in the study. Physical activity levels were assessed using the International Physical Activity Questionnaire (IPAQ), resulting in two distinct groups: 56 women in the lower activity level group and 16 in the higher activity level group. We calculated body mass index (BMI), measured body composition and blood pressure, and conducted cognitive assessments, including the Mini-Mental State Examination (MMSE), motor and psychomotor skills tests, the Clock Drawing Test, and the Geriatric Depression Scale (GDS). Nutritional intake was evaluated using a detailed 3-day food record to analyze macronutrient consumption and total caloric intake. **Results**: A statistically significant difference in total blood cholesterol levels (*p* = 0.0277) was observed between the two groups, with the higher physical activity group showing elevated cholesterol levels. Although no other biochemical markers showed statistically significant differences, variations in BDNF, glucose, triglycerides, IL-6, and insulin levels were noted between groups. Moreover, correlations between these markers and cognitive performance, like motor and psychomotor speeds, varied depending on physical activity level. The analyzed dietary pattern of the studied group shows slight deviations from current nutritional recommendations. **Conclusions**: The findings suggest that physical activity level may influence certain biochemical markers and cognitive functions in postmenopausal women. While these results highlight the potential benefits of physical activity, further research is needed to clarify underlying mechanisms and to validate physical activity as an effective strategy for improving postmenopausal health.

## 1. Introduction

Postmenopausal women are increasingly susceptible to metabolic disorders and cognitive decline, primarily due to hormonal changes, alterations in body composition, and chronic low-grade inflammation [1,2,3,4,5,6]. The menopausal transition is associated with insulin resistance, dyslipidemia, mood disturbances, and cognitive complaints, all of which significantly affect long-term health and quality of life [7,8,9,10,11,12,13].

These physiological and neuropsychological changes are largely driven by the decline in circulating estrogen levels. Estrogen deficiency promotes insulin resistance by redistributing and increasing visceral fat—impairing insulin signaling and disrupting mitochondrial energy production [14]. Simultaneously, it alters lipid metabolism, raising LDL cholesterol and triglycerides while lowering HDL cholesterol, primarily due to hepatic receptor dysregulation and reduced lipoprotein lipase activity [15,16].

Moreover, declining estrogen levels disrupt key neurotransmitter systems—especially serotonergic and dopaminergic pathways—and dysregulate the hypothalamic–pituitary–adrenal (HPA) axis, thereby increasing the risk of mood disorders such as depression and anxiety [17,18]. Estrogen also plays a crucial role in maintaining hippocampal and prefrontal cortex function; its deficiency is linked to impairments in memory, attention, and executive function [19,20]. Together, these changes significantly impact the metabolic and cognitive health of women during and after menopause.

Although mild cognitive fluctuations are common, epidemiological evidence suggests that 10–20% of postmenopausal women develop mild cognitive impairment (MCI), with a considerable proportion progressing to dementia, particularly Alzheimer’s disease [21,22]. Compared to men, women face a higher risk of neurodegenerative disorders, largely due to estrogen’s regulatory role in neurotransmission, synaptic plasticity, and neurotrophic factor expression, especially brain-derived neurotrophic factor (BDNF) [23,24,25,26,27,28].

In addition to cognitive decline, metabolic abnormalities are common among postmenopausal women. Insulin resistance affects up to 40%, and approximately 60% present with dyslipidemia, characterized by elevated total cholesterol, LDL cholesterol, and triglycerides, along with reduced HDL cholesterol [29,30]. These disturbances often coexist with elevated levels of inflammatory cytokines, particularly interleukin-6 (IL-6), which contribute to the development of cardiovascular and neurodegenerative diseases [31,32].

Biochemical markers such as glucose, insulin, lipid profile, IL-6, and BDNF are key clinical indicators of metabolic and neurological status [22,23,33]. Cognitive function in this population is commonly evaluated using standardized tools like the Mini-Mental State Examination (MMSE) and the Clock Drawing Test [34].

Although physical activity is widely recognized as essential to metabolic and cognitive health, its specific effects on biomarkers—especially BDNF and inflammatory cytokines—in postmenopausal women remain insufficiently explored [25,32,35,36,37,38,39,40,41,42,43,44]. While several interventional studies have examined the impact of physical activity on BDNF and inflammatory markers in this group, findings remain inconsistent. A meta-analysis by Szuhany et al. [45] indicated that aerobic exercise may moderately increase BDNF concentrations, whereas the effects of resistance training are less clear.

In contrast, a systematic review by Hoseinpour et al. confirmed that regular aerobic and resistance exercise significantly reduces inflammatory markers such as IL-6, TNF-α, and CRP [46]. Similarly, a study by Cheng and Yang (2024) demonstrated CRP reduction following a resistance training program in postmenopausal women [47]. However, most of the available studies are limited by small sample sizes, short durations, and insufficient control for confounding variables like hormone therapy, diet, and psychological stress. Moreover, very few studies simultaneously assess both neurotrophic and inflammatory markers, highlighting the need for more comprehensive, long-term investigations.

Physical activity contributes to metabolic regulation by improving glucose homeostasis, lipid metabolism, and inflammatory status [29,48,49,50]. Dietary components such as polyunsaturated fatty acids (PUFAs), fiber, and antioxidants also exhibit anti-inflammatory and neuroprotective effects, but their combined influence with exercise on health biomarkers in postmenopausal women remains underexplored [51,52,53,54,55,56,57,58].

The primary objective of this study was to evaluate whether varying levels of physical activity influence selected biochemical parameters (glucose, insulin, total cholesterol, triglycerides, IL-6), BDNF concentrations, and cognitive performance in postmenopausal women.

We hypothesized that women with higher levels of physical activity would demonstrate more favorable metabolic profiles, enhanced cognitive performance, and increased BDNF concentrations compared to women with lower activity levels.

Additionally, nutrient intake was assessed independently, without analyzing its interactions with other study variables.

## 2. Method

### 2.1. Participants and Ethical Aspects

This cross-sectional observational study was conducted between August and October 2020 in Gorzów Wielkopolski. The study involved 72 Caucasian women from western Poland, aged between 55 and 73 years, who were recruited through local newspaper announcements, social media, and in collaboration with the business partner REHA-MED.

Inclusion criteria for participants were: female gender, age range of 55–73 years, independence in daily activities, natural menopause (defined as the absence of menstruation for at least 12 consecutive months), non-smoking status, and absence of diagnosed chronic diseases.

Participants with surgically or treatment-induced menopause (e.g., hysterectomy or pelvic radiotherapy) and those using hormone replacement therapy (HRT) were excluded. Participants were divided into two groups based on their physical activity level determined by the International Physical Activity Questionnaire (IPAQ).

Initially, 150 women responded to the recruitment announcements and underwent a preliminary screening interview. This screening involved comprehensive questions regarding current health status, medical history, neurodegenerative disease presence, diagnosed cognitive impairments, medication use, lifestyle habits, smoking status, menopausal status, and levels of physical activity. From this group, 110 women met the initial eligibility criteria. A subsequent detailed health screening further excluded individuals with severe chronic illnesses, diagnosed cognitive disorders, neurological or psychiatric conditions, or significant metabolic or endocrine abnormalities that could influence cognitive functioning or impact study outcomes. Ultimately, 72 women who fulfilled all inclusion criteria and exhibited no contraindicating health conditions were enrolled in the study.

All procedures conducted in this research adhered strictly to the ethical guidelines outlined in the 1975 Declaration of Helsinki, as revised. The study protocol was reviewed and approved by the Bioethics Committee at Poznań University of Medical Sciences (Decision No. 989/18, dated 11.10.2018). Written informed consent was obtained from all participants prior to study enrollment.

### 2.2. Nutritional Evaluation

The participants’ dietary intake was evaluated by a certified dietitian (K.M.) using a three-day dietary recording method, as outlined by Gronowska-Senger [59]. Each participant was asked to document, in detail, all foods and beverages consumed over three consecutive days, including portion sizes and ingredients used in meal preparation.

Each participant completed the food record for two non-consecutive weekdays and one weekend day, totaling three days. To aid in portion size estimation, participants were provided with a photo album of food products and dishes [60]. The energy and nutritional value of daily food intake was calculated using the Cambridge Diagnostics computer program “Aliant.” The collected dietary data were analyzed in relation to each participant’s personalized daily energy and nutrient needs, with adjustments made for typical losses resulting from food preparation and processing. These individual requirements were estimated using the guidelines provided by the National Food and Nutrition Institute, following the recommended dietary allowances [61].

### 2.3. Anthropometric and Physical Activity Measurement

Anthropometric and body composition measurements were conducted after a 12 h fast. Body weight was assessed using a calibrated medical scale with a precision of 1 g, while height was measured with a professional medical altimeter to the nearest 0.1 cm. These values were used to compute body mass index (BMI). Additionally, body composition parameters were determined via bioelectrical impedance analysis using a Tanita MC780 Bioelectrical Impedance Analyzer.

The assessment of physical activity was conducted using the International Physical Activity Questionnaire–Short Form (IPAQ-SF). Measurements were taken in light clothing, without shoes, by the same trained personnel, according to standard techniques.

### 2.4. Assessment of Psychological Parameters and Dementia

Assessment of mental and motor functions was conducted using standardized Polish-language versions of psychological tests. Emotional state and the possible presence of depressive symptoms were evaluated with the 30-item Geriatric Depression Scale (GDS). Scores exceeding 10 points suggest mild depression, while results above 20 points indicate severe depressive symptoms [62]. Cognitive ability was assessed using the Mini-Mental State Examination (MMSE) [63], which includes a range of tasks targeting core cognitive domains. The total score ranges from 0 to 30 points. Scores between 24 and 26 may indicate cognitive impairment without dementia, while lower scores suggest varying degrees of dementia. To complement the MMSE, a modified version of the Clock Drawing Test was used, based on the method proposed by Tuokko [64], which involves completing three clock faces. Participants could score up to 3 points for correct drawings; incorrect responses were scored as 0. Motor and psychomotor speed was evaluated based on the criteria outlined in the corresponding subtests of the dementia assessment scale [65]. Motor speed was assessed by instructing participants to rapidly and widely alternate tapping between the index and middle fingers of their non-dominant hand. The result was determined by counting the number of taps performed within a 5 s period. Psychomotor speed was evaluated by asking participants to perform the following rapid sequence of three hand movements using their non-dominant hand.

(1) Form a fist on a flat surface;

(2) Place the palm flat on the surface, facing downward;

(3) Position the hand vertically on its side, resting on the edge of the little finger.

Participants were instructed to repeat the sequence as quickly and accurately as possible. Following a demonstration of the movement sequence, participants were given two practice attempts. The final score reflected the number of accurately completed sequences performed within a 10 s interval. All cognitive and motor tests (MMSE, modified Clock Drawing Test, Geriatric Depression Scale, and motor and psychomotor speed tests) were performed by trained researchers using validated Polish versions of the diagnostic tools.

### 2.5. Biochemical Parameters

Blood samples for biochemical analyses were obtained from the antecubital vein in the morning after a 12 h fast. Blood samples were collected in accordance with safety protocols using serum-separating tubes (9 mL, S-Monovette, SARSTEDT, Nümbrecht, Germany). The samples were centrifuged at 3000× *g* for 10 min at 4 °C. After centrifugation, the serum was isolated and stored at −80 °C until further analysis.

In the serum, total cholesterol (cat. No. 7-204; test sensitivity: 2.85 mg/dL; CVs: 1.61% and 3.03%), triglycerides (cat. No. 7-253; test sensitivity: 5.7 mg/dL; CVs: 1.29% and 2.15%), and glucose (cat. No. 7-201; test sensitivity: 2.8 mg/dL; CVs: 0.71% and 2.38%) concentrations were analyzed using the Accent 220S automatic biochemical analyzer (Cormay, Łomianki, Poland) and PZ Cormay S.A. (Łomianki, Poland). The serum concentrations of insulin (DRG International Inc., Springfield Township, NJ, USA; test sensitivity: 1.76 µIU/mL; CVs: 2.20% and 4.45%), IL-6 (DRG International Inc., Springfield Township, NJ, USA; test sensitivity: 2 pg/mL; CVs: 4.25% and 4.90%), and BDNF (SunRed Biological Technology, Shanghai, China, cat. No: 201-12-1303; test sensitivity: 0.05 ng/mL; CVs: <10%) were determined by immunoenzymatic assay using commercially available ELISA kits.

Systolic and diastolic blood pressure (SBP and DBP) were recorded using an automatic upper-arm monitor (Omron i-Q142, HEM-1040-E; Omron Healthcare Co., Ltd., Kyoto, Japan). Three consecutive readings were taken at 5 min intervals, in accordance with standardized procedures recommended by the International Society of Hypertension [66].

The average value of the three readings was used for analysis to minimize the influence of transient blood pressure fluctuations.

All measurements were performed by the same trained personnel to reduce inter-individual variability and ensure protocol adherence. The blood pressure monitor was calibrated prior to the study in accordance with the manufacturer’s recommendations, and its technical performance was regularly checked. Measurement data were reviewed for consistency and completeness; outliers and anomalous values were re-evaluated for potential measurement errors and protocol deviations.

### 2.6. Statistical Analysis

The collected data were processed using Statistica 13.1 software. Preliminary calculations were performed to determine basic descriptive statistics (mean, standard deviation). Then, the distribution of individual data was checked using the Shapiro–Wilk test. The results of this test guided the choice of subsequent statistical procedures, namely the analysis of relationships and differences based on the criteria adopted for dividing subjects into groups. Since most of the variables did not meet the assumption of normality, non-parametric statistical methods were applied in further analyses. For group classification, a cluster analysis model was used based on selected characteristics relevant to group differentiation. Associations between the studied variables were assessed using Spearman’s rank correlation, while group comparisons were conducted with the Mann–Whitney U test. Statistical significance was set at *p* < 0.05.

The minimum required sample size was estimated using G*Power software (version 3.1) for correlation analysis. Assuming a moderate effect size (r = 0.3), an alpha level of 0.05, a statistical power of 0.8, and a one-tailed test, the minimum sample size was calculated to be 64 participants.

The study was designed and reported in accordance with the Strengthening the Reporting of Observational Studies in Epidemiology (STROBE) guidelines for observational research.

To minimize potential sources of bias, the following measures were implemented: Selection bias was reduced by applying consistent inclusion and exclusion criteria and recruiting participants from the same target population. Information bias was limited through the use of standardized assessment protocols, with all assessments conducted by the same trained research team. Confounding was addressed in the statistical analyses by adjusting for relevant covariates such as age, body mass index (BMI), and physical activity level.

## 3. Results

A total of 150 women responded to recruitment and underwent initial screening. After applying eligibility criteria and excluding those with significant medical, neurological, or metabolic conditions, 72 women were enrolled in the study, with a mean age of approximately 61 years. The average body mass was approximately 73 kg, and the BMI index value was approximately 28, indicating that the group was classified as overweight. The average waist-to-hip ratio (WHR) was 0.9, suggesting an increased risk of cardiovascular disease. The mean systolic blood pressure (SBP) was 135 mmHg, and the mean diastolic blood pressure (DBP) was 83 mmHg. These values reflect a slightly elevated cardiovascular profile in the group.

The results of the analysis of energy and macronutrient intake for the study group (n = 72) are presented in Table 1. The obtained values were compared with the dietary reference standards established for the Polish population [67]. The mean daily energy intake was 1722 kcal, representing 77% of the recommended intake (2000–2500 kcal). The median was 1656 kcal (range: 996–3143 kcal).

The mean protein intake was 80 g, representing 128% of the recommended intake (50–75 g). The median was 76 g (range: 40–151 g). The mean fat intake was 64 g, covering 91% of the recommended range (60–80 g). The median was 61 g (range: 29–139 g). The mean intake of saturated fatty acids (SFAs) was 24 g, potentially exceeding the recommended intake (<10% of total energy). The mean intake of monounsaturated fatty acids (MUFAs) was 23 g, while polyunsaturated fatty acids (PUFAs) averaged 11 g. The mean carbohydrate intake was 214 g, representing 76% of the recommended intake (260–300 g). The median was 215 g (range: 46–394 g). The mean dietary fiber intake was 23 g, covering 71% of the recommended range (25–40 g). The median was 22 g (range: 7–54 g). The mean cholesterol intake was 331 mg, with a median of 326 mg (range: 101–658 mg).

Cluster analysis was used to determine the grouping of women into subgroups, allowing for the identification of the optimal division based on the introduced criteria. Cluster analysis grouped participants based on the MET values of three variables from the IPAQ. This division considered three types of physical exertion: intense physical effort, moderate physical effort, and walking. Both groups were selected to ensure significant differences across the selected parameters. Table 2 presents the results, where Group A (n = 56) represents individuals with lower physical activity, while Group B (n = 16) represents women with higher activity.

A comparison of selected anthropometric parameters between the two groups is presented in Table 3. Women with lower physical activity had a higher BMI (29) than those with higher physical activity (27). There were statistically significant differences in waist circumference (97 cm and 90 cm, respectively), as well as in the WHR ratio (0.91 and 0.87, respectively).

Table 4 presents a comparison of cognitive and biochemical parameters in the groups with lower (Group A) and higher (Group B) physical activity. Statistically significant differences were observed in blood cholesterol levels (*p* = 0.0277), with Group A having an average level of approximately 194 and Group B approximately 223 mg/dL.

Table 5 presents correlations in Group A between cholesterol content and intense physical exercise (r = 0.2806) and psychomotor performance and moderate physical exercise (r = 0.3822). Moreover, there were positive correlations between BDNF and intense and moderate physical exercise (r = 0.3275 and r = 0.3877, respectively).

Table 6 shows the correlations observed in Group B between cholesterol content and intense physical exercise (r = 0.2561) and walking (r = −0.2387). BDNF levels were correlated with moderate physical exercise (r= −0.3683) and walking (r = 0.3335). Moreover, there were negative correlations between IL-6 and insulin in intense physical exercise (r = −02844 and r = −0.3707, respectively). There was a positive relationship between BDNF levels and cholesterol in Group A and a significant negative relationship between BDNF levels and glucose in Group B.

Table 7 presents correlation coefficients between BDNF and cholesterol, glucose, IL-6, triglycerides, and insulin in Group A (n = 56). Statistically significant positive correlations were observed between BDNF and cholesterol (r = 0.3462, *p* = 0.0089). The same set of biochemical parameters was analyzed in Group B (n = 16). A significant negative correlation was found between BDNF and glucose (r = −0.7452, *p* = 0.0009), indicating an inverse relationship.

## 4. Discussion

Our study demonstrated that higher physical activity levels in postmenopausal women were associated with favorable biochemical and cognitive outcomes. Women in the higher physical activity group exhibited significantly higher total cholesterol levels but also show more favorable profiles for glucose, triglyceride, insulin, and BDNF levels. Moreover, moderate and vigorous physical activity were positively associated with improved cognitive function, underscoring the importance of physical activity in maintaining neurocognitive health. These findings reveal the complex interplay between physical activity, metabolic health, and cognitive function and support the need for further targeted research in this population. Future studies should incorporate larger cohorts and longitudinal and/or interventional designs to establish causality and clarify underlying biological mechanisms.

Aging is associated with a decline in both cognitive and physiological functions, leading to an increased prevalence of chronic diseases and functional impairment. These health conditions are a major contributor to reduced independence, limiting the ability of older adults to perform everyday tasks [27,71]. Lifestyle factors, particularly physical activity, play a crucial role in mitigating these age-related declines. Physical activity has been shown to confer neuroprotective, immunomodulatory, and metabolic benefits [72,73,74].

Our findings indicate that Group B, characterized by higher total cholesterol levels, exhibited a tendency toward lower triglyceride concentrations compared to Group A, although this difference did not reach statistical significance. Triglyceride levels were negatively correlated with walking in Group A, while in Group B, a negative correlation was observed between triglycerides and vigorous physical activity.

According to Table 5, the group with higher physical activity exhibited elevated total cholesterol levels, which is an unexpected finding, given that physical activity is generally associated with favorable lipid profile modulation. One explanation is the increase in high-density lipoprotein (HDL) cholesterol induced by regular physical exercise. Since total cholesterol (TC) comprises LDL, HDL, and VLDL fractions, an HDL-related rise in TC may not reflect adverse lipid status or negatively impact cardiovascular health. Elevated HDL levels are particularly beneficial in postmenopausal women [75,76]. Therefore, future studies should monitor both total cholesterol and its subfractions, especially, to improve cardiovascular risk assessment.

Cholesterol levels can also be influenced by factors such as diet, training-induced metabolic adaptations, and genetic background. Some evidence suggests that exercise may transiently increase TC due to lipid mobilization and redistribution, which are later balanced by improved lipid clearance and reverse transport [77,78,79,80,81,82].

A negative correlation between triglycerides and vigorous activity supports improved skeletal muscle lipid oxidation. These findings align with previous studies Początek formularzaDół formularza [83], and future research should explore lipid subfractions (e.g., LDL particle size, HDL functionality) to provide more detailed insight into cardiovascular risk.

Regular, structured physical activity is a key factor in metabolic regulation [84]. In our study, we observed significant correlations between physical activity and glucose metabolism. In Group A, intense physical effort was negatively correlated with blood glucose levels. In Group B, walking was negatively correlated with glucose levels, while intense physical activity was inversely associated with interleukin 6 (IL-6) and insulin levels.

These findings align with existing literature indicating that aerobic exercise improves insulin sensitivity by modulating glucose uptake and reducing systemic inflammation. At the onset of light-to-moderate aerobic activity, glucagon secretion increases while insulin release decreases. During higher-intensity exercise, insulin initially drops but subsequently rebounds during recovery to restore homeostasis [85,86].

The observed negative correlation between high-intensity physical activity, prolonged walking (averaging 1113 min per week), and glucose levels reinforces the recommendation of structured aerobic training as a non-pharmacological approach to improving glucose metabolism and reducing inflammation, particularly in postmenopausal women.

Both resistance and aerobic exercise trigger neurotrophic factor release, including BDNF and IGF-1, which support adult neuroplasticity [87,88,89]. Plasma BDNF is associated with metabolic syndrome and cardiovascular risk [90]. Exercise-induced increases in BDNF enhance cognition and mood [43].

In our study, moderate and intense physical activity in Group A were positively correlated with BDNF levels, whereas in Group B, walking was associated with increased BDNF levels, while moderate-intensity activity was negatively correlated. These findings suggest that different levels of exercise may differentially modulate neurotrophic responses.

Previous studies have shown that sustained physical training over weeks to months leads to an elevation in resting BDNF levels compared to pre-training value [91,92,93,94]. This effect is dependent on both exercise intensity and duration [95,96]. Exercise-induced BDNF production occurs in neuronal cells, where it is temporarily stored before being released into circulation. Peripheral BDNF can then be absorbed by both central and peripheral tissues, facilitating neuroprotective and neurotrophic processes. This cycle may explain the transient elevations in BDNF levels observed during and after exercise [91,97].

Regular physical activity is a significant protective factor against cognitive impairment and dementia in older adults. Exercise serves as a critical non-pharmacological intervention for cognitive disorders and neurodegenerative diseases, enhancing cognitive resilience [98].

Cognitive health is strongly linked to aerobic capacity, moderate-to-vigorous physical activity, and reduced sedentary behavior. The WHO recommends 150–300 min of moderate-intensity or 75–150 min of high-intensity physical activity weekly, along with muscle-strengthening exercises at least twice per week [77].

Our results confirm previous findings: Walking was negatively correlated with GDS scores, and moderate activity was associated with improvements in motor and psychomotor speed. In Group A, intense physical effort was linked to enhanced psychomotor performance and reduced depressive symptoms. In Group B, walking and intense exercise were associated with better outcomes in cognitive assessments, including performance in the Clock Drawing Test.

A meta-analysis by Colcombe et al. [99] confirmed that physical exercise enhances multiple cognitive domains, including information processing speed, spatial cognition, and executive control. Ekblom et al. [100] demonstrated that moderate-to-vigorous physical activity, measured using accelerometers, was associated with improved verbal abilities, while higher aerobic fitness correlated with superior executive attention and processing speed. Additionally, Nakagawa et al. [101] found that high-intensity exercise was linked to better mental health and cognitive function compared to low-intensity activity.

Our findings suggest that sustained lower-intensity activity, such as walking (averaging 1113 min per week), is positively associated with cognitive function, reinforcing the notion that regular physical engagement, regardless of intensity, contributes to cognitive preservation and enhancement in postmenopausal women.

Furthermore, our data show that physical activity levels also correlate with BDNF levels, suggesting a mechanistic link between physical engagement and neuroplasticity. In the group of women with lower physical activity levels, we observed positive correlations between BDNF and both moderate (r = 0.3877) and high-intensity exercise (r = 0.3275), indicating that even moderate increases in activity can induce beneficial neuroplastic adaptations. Among women with higher physical activity, elevated BDNF levels were significantly associated with walking, possibly reflecting long-term neuronal adaptation to low-intensity exercise [32,44].

Biological mechanisms underlying this association are well supported in the literature. Regular physical activity activates the PGC-1α–FNDC5 signaling pathway, resulting in the release of the myokine irisin. Irisin crosses the blood–brain barrier and stimulates the expression of the *BDNF* gene in hippocampal neurons—a key region involved in learning and memory [102]. Furthermore, exercise increases levels of β-hydroxybutyrate (β-HB), a metabolic by-product that promotes epigenetic upregulation of *BDNF* through histone acetylation [43].

Other contributing factors include improved cerebral perfusion, enhanced delivery of oxygen and glucose to neurons, and suppression of inflammatory cytokines (e.g., IL-6) and cortisol, all of which support *BDNF* expression and reduce neurotoxicity [29].

This effect likely depends on both exercise intensity and duration. Systematic reviews and meta-analyses have shown that chronic aerobic training (lasting ≥6–12 weeks) leads to sustained increases in resting *BDNF* levels in both blood and cerebrospinal fluid [45]. This mechanism is especially relevant in postmenopausal women, as declining estrogen levels have been shown to reduce endogenous *BDNF* production and increase the risk of neurodegeneration [103].

Our findings support this mechanistic model, providing evidence that regular physical activity—regardless of intensity—can effectively enhance brain neuroplasticity via increased *BDNF* levels in postmenopausal women. The observed correlations between physical exertion and *BDNF* suggest that exercise may function as a natural neuromodulator, improving cognitive health in this vulnerable population.

Nutrient intake is also crucial for postmenopausal health. Although dietary habits in this study did not deviate significantly from recommendations, some imbalances were noted. Energy intake was slightly below recommendations (1722 ± 437 kcal, ~77%), which may affect metabolic function and muscle preservation [104].

Protein intake (80 g/day) exceeded reference levels, supporting muscle maintenance, but excess—especially from animal sources—may carry metabolic risks [105,106,107,108].

Saturated fat intake (24 g/day) was relatively high, raising concerns about elevated LDL levels and cardiovascular risk [109,110,111,112,113].

Carbohydrate intake was lower than the recommended range, averaging 214 g (76% of the recommended 260–300 g). While moderate carbohydrate restriction may be beneficial for glycemic control, chronically insufficient intake may affect cognitive function and energy levels, particularly in older individuals. Further analysis of the balance between complex and simple carbohydrates is necessary to ensure adequate fiber intake and glycemic stability [114,115,116,117].

Dietary fiber intake averaged 23 g, which accounted for approximately 71% of the recommended level (25–40 g). Given the importance of fiber in digestive health, glycemic control, and cardiovascular disease prevention, efforts should be made to increase its intake. The diet should prioritize whole grains, legumes, fruits, and vegetables to ensure adequate fiber consumption [52,53,118,119].

Cholesterol intake averaged 331 mg, slightly exceeding the traditionally recommended upper limit of 300 mg. Although recent dietary guidelines have placed less emphasis on strict cholesterol restriction, dietary patterns rich in cholesterol-containing foods should be monitored, particularly in individuals at increased risk of cardiovascular disease [120,121,122,123].

This study is not without limitations. First, the sample size is relatively small, which may limit the generalizability of the findings. However, despite this limitation, the study design allowed for statistically significant conclusions regarding the impact of physical activity on biochemical markers, brain-derived neurotrophic factor (BDNF) levels, and cognitive functions in postmenopausal women. Future studies should include larger and more diverse cohorts to further validate and expand these findings.

Second, the cross-sectional nature of the study prevents the establishment of causal relationships. While the observed associations provide valuable insights, prospective longitudinal or interventional studies are necessary to determine the long-term effects of physical activity on metabolic and cognitive health in postmenopausal women.

Third, physical activity levels were assessed using the validated International Physical Activity Questionnaire–Short Form (IPAQ-SF). However, future research should use more objective methods, such as accelerometry, to enhance measurement accuracy.

Despite these limitations, this study provides significant evidence on the relationships between physical activity levels, biochemical parameters, and cognitive functions in postmenopausal women, emphasizing the need for further research in this area. Moreover, these findings contribute to the long-term accumulation of knowledge regarding women’s health.

## 5. Conclusions

This cross-sectional study demonstrated that higher levels of physical activity in postmenopausal women are associated with more favorable metabolic parameters—specifically, lower concentrations of glucose, insulin, and triglycerides—as well as higher levels of brain-derived neurotrophic factor (BDNF) and enhanced cognitive performance. These results confirm the critical role of physical activity as a modulator of metabolic and neurocognitive health during the postmenopausal period. The observed associations may be mediated by biological mechanisms such as improved glucose regulation, reduced systemic inflammations, and enhanced neurotrophic signaling pathways, including BDNF-related processes. The observed associations highlight the necessity of incorporating regular physical activity—particularly of moderate to vigorous intensity—into preventive strategies aimed at reducing age-related metabolic and cognitive disorders in women.

Public health initiatives should consider implementing exercise programs tailored to the specific needs of postmenopausal women, and healthcare professionals are encouraged to routinely assess physical activity levels as part of risk evaluation in this population.

While the cross-sectional design precludes causal inference, these results support further longitudinal and interventional studies to clarify underlying mechanisms and long-term effects.

In light of these findings, physical activity emerges as a promising non-pharmacological intervention that promotes healthy aging, cognitive resilience, and improved metabolic function in postmenopausal women.

## Figures and Tables

**Table 1:** Comparison of macronutrient consumption in the study group (n = 72).

Variable	Total (n = 72)		
	Arithmetic Mean ± sd Median (Min-Max)	References Rda	(%)
Total caloric intake (kcal)	1722 ± 437 1656 (996–3143)	2000–2500 kcal/person/day	77
Total protein (g)	80 ± 22 76 (40–151)	50–75 g/person/day	128
Protein daily intake (g/kg body weight)	1.09	0.9 g/kg body weight	121
Total fat (g)	64 ± 21 61 (29–139)	60–80 g/person/day	91
Total carbohydrates (g)	214 ± 65 215 (46–394)	260–300 g/person/day	76
Dietary fiber (g)	23 ± 8 22 (7–54)	25–40 g/person/day	71
Plant protein (g)	23 ± 8 21 (5–56)	no clear standards	-
Animal protein (g)	52 ± 19 50 (21–104)	no clear standards	-
SFA (g)	24 ± 16 23 (9–118)	<10% energy	over 10%
MUFA (g)	23 ± 8 22 (10–49)	10–15% energy	-
PUFA (g)	11 ± 5 10 (4–38)	6–8% energy	-
Cholesterol (mg)	331 ± 131 326 (101–658)	>300 mg	110

SFA: saturated fatty acid; MUFA: monounsaturated fatty acid; PUFA: polyunsaturated fatty acid; RDA: recommended dietary allowance (the recommended intake level as applied in dietary standards for the Polish population) [67].

**Table 2:** Division into groups of women with different physical activity.

Variable	Lower Physical Activity Group (Group A) (n = 56)	Higher Physical Activity Group (Group B) (n = 16)	Test U M-W *p*
	Arithmetic Mean ± SD		
Total MET min/week	2848.0 ± 2772.3	8210.4 ± 4234.7	<0.001

MET: metabolic equivalent of task

**Table 3:** A comparison of selected anthropometric parameters in the lower physical activity group (Group A) and higher physical activity group (Group B).

Variable	Lower Physical Activity Group (Group A) (n = 56)	Higher Physical Activity Group (Group B) (n = 16)	
	Arithmetic Mean ± SD		Test M-W *p*
BMI (kg/m^2^)	29 ± 5	27 ± 6	0.0553
WC (cm)	97 ± 11	90 ± 15	**0.0136**
WHR	0.91 ± 0.07	0.87 ± 0.11	**0.0138**
SBP (mmHg)	135 ± 19	132 ± 13	0.9622
DBP (mmHg)	84 ± 15	82 ± 12	0.6892
PULSE	73 ± 8	73 ± 9	0.7345

BMI: body mass index; WC: waist circumference; WHR: waist–hip ratio; SBP: systolic blood pressure; DBP: diastolic blood pressure. All the *p*-values marked in bold are statistically significant (*p* < 0.05).

**Table 4:** A comparison of cognitive and biochemical parameters in the lower physical activity group (Group A) and higher physical activity group (Group B).

Variable	Lower Physical Activity Group (Group A) (n = 56)	Higher Physical Activity Group (Group B) (n = 16)	References	Test M-W *p*
GDS (points)	4.11 ± 5.20	5.19 ± 7.39	0–9—no depression 10–19—mild depression above 20–deep depression [62]	0.9235
Motor speed	22.47 ± 3.42	23.06 ± 2.29	no clear standards	0.6448
Psychomotor speed	5.66 ± 1.91	6.26 ± 2.51	no clear standards	0.5369
MMSE (points)	28.73 ± 1.77	28.44 ± 1.26	30–27—normal result 26–24—cognitive impairment without dementia 23–19—mild dementia 18–11—moderate dementia 10–0—profound dementia [63]	0.1563
Clock Drawing Test (points)	2.75 ± 0.51	2.94 ± 0.25	0–3 [64]	0.1649
Glucose (mg/dL)	**106.77 ± 21.89**	97.13 ± 13.16	<100 [68]	0.0569
Triglycerides (mg/dL)	**233.38 ± 135.25**	**157.88 ± 62.47**	<100 [69]	0.0645
Cholesterol (mg/dL)	**194.05 ± 46.25**	**223.31 ± 47.41**	<190 [69]	**0.0277**
BDNF (ng/mL)	1.81 ± 1.21	2.26 ± 1.05	no clear standards	0.0714
Interleukin IL-6 (pg/mL)	**25.60 ± 37.91**	**79.39 ± 248.80**	<6 [70]	0.4281
Insulin (μLU/mL)	17.33 ± 11.39	14.10 ± 9.03	3–17 [68]	0.1530

GDS: Geriatric Depression Scale; MMSE: Mini-Mental State Examination; BDNF: brain-derived neurotrophic factor. All the *p*-values marked in bold are statistically significant (*p* < 0.05).

**Table 5:** Correlation analyses for Group A (women with lower physical activity).

Group A (n = 56)	Intensive Physical Effort (Minutes/Week)	Moderate Physical Effort (Minutes/Week)	Walking (Minutes/Week)
BMI (kg/m^2^) r *p*	−0.0045 0.9843	0.1085 0.5027	−0.0514 0.4670
SBP (mmHg) r *p*	−0.1750 0.2203	−0.0639 0.6242	0.0561 0.8161
DBP (mmHg) r *p*	−0.1821 0.2076	0.1176 0.5560	0.0822 0.9739
PULSE (bpm) r *p*	−0.0059 0.9358	0.1779 0.2373	−0.1632 0.2036
WC (cm) r *p*	0.0227 0.2520	0.0718 0.0825	−0.1390 0.3546
WHR r p	−0.0366 0.8206	−0.1498 0.2796	−0.1746 0.2114
Glucose (mg/dL) r *p*	**−0.2416** **0.0419**	−0.0161 0.9377	−0.1754 0.3011
Triglycerides (mg/dL) r *p*	−0.0843 0.5305	0.0466 0.6034	**−0.2028** **0.0425**
Cholesterol (mg/dL) r *p*	**0.2806** **0.0436**	0.1742 0.1594	−0.0029 0.9245
GDS (points) r *p*	**−0.2044** **0.0482**	**−0.2539** **0.0490**	0.0323 0.9173
Motor speed r *p*	0.1647 0.1547	**0.2485** **0.0467**	0.0525 0.7441
Psychomotor speed r *p*	**0.2598** **0.0487**	**0.3822** **0.0099**	−0.1554 0.2309
MMSE (points) r *p*	−0.0055 0.7952	0.0737 0.4520	−0.0832 0.4716
Clock Drawing Test (points) r *p*	0.1488 0.3401	0.1192 0.3381	**−0.2098** **0.0473**
BDNF (ng/mL) r *p*	**0.3275** **0.0389**	**0.3877** **0.0489**	−0.0201 0.7349
Interleukin IL-6 (pg/mL) r *p*	−0.0832 0.3589	−0.0916 0.9389	−0.1168 0.7020
Insulin (μLU/mL) r *p*	−0.1794 0.1640	−0.0718 0.7776	−0.1020 0.2200

BMI: body mass index; SBP: systolic blood pressure; DBP: diastolic blood pressure; WC: waist circumference; WHR: waist–hip ratio; GDS: Geriatric Depression Scale; MMSE: Mini-Mental State Examination; BDNF: brain-derived neurotrophic factor. All the *p*-values marked in bold are statistically significant (*p* < 0.05).

**Table 6:** Correlation analyses for Group B (women with higher physical activity).

Group B (n = 16)	Intensive Physical Activity (Minutes/Week)	Moderate Physical Activity (Minutes/Week)	Walking (Minutes/Week)
BMI (kg/m^2^) r *p*	**−0.4106** **0.0424**	−0.0872 0.7349	−0.1186 0.6983
SBP (mmHg) r *p*	−0.0733 0.7007	**0.4138** **0.0411**	0.0000 0.9951
DBP (mmHg) r *p*	−0.1884 0.4016	0.1968 0.4095	0.1621 0.4698
PULSE **(bpm)** r *p*	0.0594 0.8983	0.0203 0.7355	**−0.2612** **0.0465**
WC (cm) r *p*	**−0.4843** **0.0201**	0.0848 0.0687	0.1453 0.5378
WHR r *p*	**−0.3887** **0.0389**	0.0538 0.7771	**0.3974** **0.0398**
Glucose (mg/dL) r *p*	−0.1144 0.6623	0.1555 0.4589	**−0.2962** **0.0377**
Triglycerides (mg/dL) r *p*	**−0.2963** **0.0389**	−0.1203 0.6442	−0.0865 0.7826
Cholesterol (mg/dL) r *p*	**0.2561** **0.476**	0.0283 0.8913	**−0.2387** **0.0421**
GDS (points) r *p*	0.1173 0.7904	0.0498 0.7969	**−0.2558** **0.0401**
Motor speed r *p*	−0.1205 0.6992	**−0.2288** **0.0376**	**0.3769** **0.0277**
Psychomotor speed r *p*	0.1477 0.6433	−0.0430 0.8816	−0.1379 0.6021
MMSE (points) r *p*	−0.1428 0.4647	0.0115 0.8350	0.1340 0.5256
Clock Drawing Test (points) r *p*	**0.4098** **0.0456**	0.0000 0.9987	0.0000 0.9876
BDNF (ng/mL) r *p*	0.0492 0.5827	**−0.3683** **0.0497**	**0.3335** **0.0434**
Interleukin IL-6 (pg/mL) r *p*	**−0.2844** **0.0401**	0.0012 0.8005	0.0000 0.9942
Insulin (μLU/mL) r *p*	**−0.3707** **0.0387**	0.1301 0.8195	0.0321 0.9279

BMI: body mass index; SBP: systolic blood pressure; DBP: diastolic blood pressure; WC: waist circumference; WHR: waist–hip ratio; GDS: Geriatric Depression Scale; MMSE: Mini-Mental State Examination; BDNF: brain-derived neurotrophic factor. All the *p*-values marked in bold are statistically significant (*p* < 0.05).

**Table 7:** Spearman correlations among BDNF and biochemical parameters in Groups A and B.

Biochemical Parameters	BDNF Group A (n = 56)	BDNF Group B (n = 16)
Cholesterol (mg/dL) r *p*	**0.3462** **0.0089**	0.1384 0.6012
Glucose (mg/dL) r *p*	0.1165 0.3886	**−0.7452** **0.0009**
IL-6 (pg/mL) r *p*	0.1229 0.3629	−0.2064 0.4413
Triglycerides r *p*	0.1613 0.2343	−0.2373 0.3759
Insulin (uLU/mL) r *p*	0.0648 0.6401	−0.0386 0.8834

BDNF: brain-derived neurotrophic factor. All the *p*-values marked in bold are statistically significant (*p* < 0.05).

## Data Availability

The datasets generated and/or analyzed during the current study are not publicly available because the data were obtained without consent for sharing, but they are available from the corresponding author on reasonable request.

## References

[1] Rizzo M.R., Fasano R., Paolisso G. (2020). Adiponectin and Cognitive Decline. Int. J. Mol. Sci..

[2] Wang Y., Mishra A., Brinton R.D. (2020). Transitions in Metabolic and Immune Systems from Pre-Menopause to Post-Menopause: Implications for Age-Associated Neurodegenerative Diseases. F1000Research.

[3] Jeong H.G., Park H. (2022). Metabolic Disorders in Menopause. Metabolites.

[4] Sochocka M., Karska J., Pszczołowska M., Ochnik M., Fułek M., Fułek K., Kurpas D., Chojdak-Łukasiewicz J., Rosner-Tenerowicz A., Leszek J. (2023). Cognitive Decline in Early and Premature Menopause. Int. J. Mol. Sci..

[5] Genazzani A.D., Petrillo T., Semprini E., Aio C., Foschi M., Ambrosetti F., Sponzilli A., Ricciardiello F., Battipaglia C. (2024). Metabolic Syndrome, Insulin Resistance and Menopause: The Changes in Body Structure and the Therapeutic Approach. Gynecol. Reprod. Endocrinol. Metab..

[6] Motlani V., Motlani G., Pamnani S., Sahu A., Acharya N. (2023). Changed Endocrinology in Postmenopausal Women: A Comprehensive View. Cureus.

[7] Avis N.E., Crawford S.L., Greendale G., Bromberger J.T., Everson-Rose S.A., Gold E.B., Hess R., Joffe H., Kravitz H.M., Tepper P.G. (2015). Duration of Menopausal Vasomotor Symptoms Over the Menopause Transition. JAMA Intern. Med..

[8] Bromberger J.T., Kravitz H.M., Chang Y.-F., Cyranowski J.M., Brown C., Matthews K.A. (2011). Major Depression during and after the Menopausal Transition: Study of Women’s Health Across the Nation (SWAN). Psychol. Med..

[9] El Khoudary S.R., Greendale G., Crawford S.L., Avis N.E., Brooks M.M., Thurston R.C., Karvonen-Gutierrez C., Waetjen L.E., Matthews K. (2019). The Menopause Transition and Women’s Health at Midlife: A Progress Report from the Study of Women’s Health Across the Nation (SWAN). Menopause.

[10] Hearing C.M., Chang W.C., Szuhany K.L., Deckersbach T., Nierenberg A.A., Sylvia L.G. (2016). Physical Exercise for Treatment of Mood Disorders: A Critical Review. Curr. Behav. Neurosci. Rep..

[11] Kravitz H.M., Janssen I., Bromberger J.T., Matthews K.A., Hall M.H., Ruppert K., Joffe H. (2017). Sleep Trajectories Before and After the Final Menstrual Period in the Study of Women’s Health Across the Nation (SWAN). Curr. Sleep Med. Rep..

[12] Mark J.K.K., Samsudin S., Looi I., Yuen K.H. (2024). Vaginal Dryness: A Review of Current Understanding and Management Strategies. Climacteric J. Int. Menopause Soc..

[13] Tepper P.G., Brooks M.M., Randolph J.F.J., Crawford S.L., El Khoudary S.R., Gold E.B., Lasley B.L., Jones B., Joffe H., Hess R. (2016). Characterizing the Trajectories of Vasomotor Symptoms across the Menopausal Transition. Menopause.

[14] Mauvais-Jarvis F. (2011). Estrogen and Androgen Receptors: Regulators of Fuel Homeostasis and Emerging Targets for Diabetes and Obesity. Trends Endocrinol. Metab. TEM.

[15] Nie G., Yang X., Wang Y., Liang W., Li X., Luo Q., Yang H., Liu J., Wang J., Guo Q. (2022). The Effects of Menopause Hormone Therapy on Lipid Profile in Postmenopausal Women: A Systematic Review and Meta-Analysis. Front. Pharmacol..

[16] Uddenberg E.R., Safwan N., Saadedine M., Hurtado M.D., Faubion S.S., Shufelt C.L. (2024). Menopause Transition and Cardiovascular Disease Risk. Maturitas.

[17] Joffe H., de Wit A., Coborn J., Crawford S., Freeman M., Wiley A., Athappilly G., Kim S., Sullivan K.A., Cohen L.S. (2020). Impact of Estradiol Variability and Progesterone on Mood in Perimenopausal Women With Depressive Symptoms. J. Clin. Endocrinol. Metab..

[18] Herrera-Pérez J.J., Hernández-Hernández O.T., Flores-Ramos M., Cueto-Escobedo J., Rodríguez-Landa J.F., Martínez-Mota L. (2024). The Intersection between Menopause and Depression: Overview of Research Using Animal Models. Front. Psychiatry.

[19] Conde D.M., Verdade R.C., Valadares A.L.R., Mella L.F.B., Pedro A.O., Costa-Paiva L. (2021). Menopause and Cognitive Impairment: A Narrative Review of Current Knowledge. World J. Psychiatry.

[20] Pertesi S., Coughlan G., Puthusseryppady V., Morris E., Hornberger M. (2019). Menopause, Cognition and Dementia—A Review. Post Reprod. Health.

[21] Beam C.R., Kaneshiro C., Jang J.Y., Reynolds C.A., Pedersen N.L., Gatz M. (2018). Differences Between Women and Men in Incidence Rates of Dementia and Alzheimer’s Disease. J. Alzheimers Dis. JAD.

[22] Ko S.-H., Jung Y. (2021). Energy Metabolism Changes and Dysregulated Lipid Metabolism in Postmenopausal Women. Nutrients.

[23] Ko S.-H., Kim H.-S. (2020). Menopause-Associated Lipid Metabolic Disorders and Foods Beneficial for Postmenopausal Women. Nutrients.

[24] Shan C., Zhang C., Zhang C. (2024). The Role of IL-6 in Neurodegenerative Disorders. Neurochem. Res..

[25] Smith P.J., Blumenthal J.A., Hoffman B.M., Cooper H., Strauman T.A., Welsh-Bohmer K., Browndyke J.N., Sherwood A. (2010). Aerobic Exercise and Neurocognitive Performance: A Meta-Analytic Review of Randomized Controlled Trials. Psychosom. Med..

[26] Nascimento C.M.C., Pereira J.R., de Andrade L.P., Garuffi M., Talib L.L., Forlenza O.V., Cancela J.M., Cominetti M.R., Stella F. (2014). Physical Exercise in MCI Elderly Promotes Reduction of Pro-Inflammatory Cytokines and Improvements on Cognition and BDNF Peripheral Levels. Curr. Alzheimer Res..

[27] Christensen A., Pike C.J. (2015). Menopause, Obesity and Inflammation: Interactive Risk Factors for Alzheimer’s Disease. Front. Aging Neurosci..

[28] Hara Y., Waters E.M., McEwen B.S., Morrison J.H. (2015). Estrogen Effects on Cognitive and Synaptic Health Over the Lifecourse. Physiol. Rev..

[29] Khalafi M., Malandish A., Rosenkranz S.K. (2021). The Impact of Exercise Training on Inflammatory Markers in Postmenopausal Women: A Systemic Review and Meta-Analysis. Exp. Gerontol..

[30] Svensson M., Lexell J., Deierborg T. (2015). Effects of Physical Exercise on Neuroinflammation, Neuroplasticity, Neurodegeneration, and Behavior: What We Can Learn From Animal Models in Clinical Settings. Neurorehabil. Neural Repair.

[31] Whitmer R.A., Gunderson E.P., Barrett-Connor E., Quesenberry C.P., Yaffe K. (2005). Obesity in Middle Age and Future Risk of Dementia: A 27 Year Longitudinal Population Based Study. BMJ.

[32] Leckie R.L., Oberlin L.E., Voss M.W., Prakash R.S., Szabo-Reed A., Chaddock-Heyman L., Phillips S.M., Gothe N.P., Mailey E., Vieira-Potter V.J. (2014). BDNF Mediates Improvements in Executive Function Following a 1-Year Exercise Intervention. Front. Hum. Neurosci..

[33] Colucci-D’Amato L., Speranza L., Volpicelli F. (2020). Neurotrophic Factor BDNF, Physiological Functions and Therapeutic Potential in Depression, Neurodegeneration and Brain Cancer. Int. J. Mol. Sci..

[34] Arevalo-Rodriguez I., Smailagic N., Roqué-Figuls M., Ciapponi A., Sanchez-Perez E., Giannakou A., Pedraza O.L., Bonfill Cosp X., Cullum S. (2021). Mini-Mental State Examination (MMSE) for the Early Detection of Dementia in People with Mild Cognitive Impairment (MCI). Cochrane Database Syst. Rev..

[35] Anderson D., Seib C., Rasmussen L. (2014). Can Physical Activity Prevent Physical and Cognitive Decline in Postmenopausal Women? A Systematic Review of the Literature. Maturitas.

[36] Baker A., Sirois-Leclerc H., Tulloch H. (2016). The Impact of Long-Term Physical Activity Interventions for Overweight/Obese Postmenopausal Women on Adiposity Indicators, Physical Capacity, and Mental Health Outcomes: A Systematic Review. J. Obes..

[37] Gheysen F., Poppe L., DeSmet A., Swinnen S., Cardon G., De Bourdeaudhuij I., Chastin S., Fias W. (2018). Physical Activity to Improve Cognition in Older Adults: Can Physical Activity Programs Enriched with Cognitive Challenges Enhance the Effects? A Systematic Review and Meta-Analysis. Int. J. Behav. Nutr. Phys. Act..

[38] Northey J.M., Cherbuin N., Pumpa K.L., Smee D.J., Rattray B. (2018). Exercise Interventions for Cognitive Function in Adults Older than 50: A Systematic Review with Meta-Analysis. Br. J. Sports Med..

[39] Zhu X., Yin S., Lang M., He R., Li J. (2016). The More the Better? A Meta-Analysis on Effects of Combined Cognitive and Physical Intervention on Cognition in Healthy Older Adults. Ageing Res. Rev..

[40] Barha C.K., Falck R.S., Davis J.C., Nagamatsu L.S., Liu-Ambrose T. (2017). Sex Differences in Aerobic Exercise Efficacy to Improve Cognition: A Systematic Review and Meta-Analysis of Studies in Older Rodents. Front. Neuroendocrinol..

[41] Karssemeijer E.G.A., Aaronson J.A., Bossers W.J.R., Donders R., Olde Rikkert M.G.M., Kessels R.P.C. (2019). The Quest for Synergy between Physical Exercise and Cognitive Stimulation via Exergaming in People with Dementia: A Randomized Controlled Trial. Alzheimers Res. Ther..

[42] Loprinzi P.D. (2019). The Role of Astrocytes on the Effects of Exercise on Episodic Memory Function. Physiol. Int..

[43] Sleiman S.F., Henry J., Al-Haddad R., El Hayek L., Abou Haidar E., Stringer T., Ulja D., Karuppagounder S.S., Holson E.B., Ratan R.R. (2016). Exercise Promotes the Expression of Brain Derived Neurotrophic Factor (BDNF) through the Action of the Ketone Body β-Hydroxybutyrate. eLife.

[44] Zoladz J.A., Pilc A. (2010). The Effect of Physical Activity on the Brain Derived Neurotrophic Factor: From Animal to Human Studies. J. Physiol. Pharmacol. Off. J. Pol. Physiol. Soc..

[45] Szuhany K.L., Bugatti M., Otto M.W. (2015). A Meta-Analytic Review of the Effects of Exercise on Brain-Derived Neurotrophic Factor. J. Psychiatr. Res..

[46] Nejatian Hoseinpour A., Bassami M., Ahmadizad S., Donath L., Setayesh S., Mirzaei M., Mohammad Rahimi G.R. (2025). The Influence of Resistance Training on Inflammatory Markers, Body Composition and Functional Capacity in Healthy Older Adults: A Systematic Review and Meta-Analysis. Arch. Gerontol. Geriatr..

[47] Cheng X., Yang Z. (2024). Effect of Resistance Training on Inflammatory Markers in Middle-Aged and Older Adults: A Meta-Analysis. Arch. Gerontol. Geriatr..

[48] Casaletto K.B., Lindbergh C.A., VandeBunte A., Neuhaus J., Schneider J.A., Buchman A.S., Honer W.G., Bennett D.A. (2022). Microglial Correlates of Late Life Physical Activity: Relationship with Synaptic and Cognitive Aging in Older Adults. J. Neurosci. Off. J. Soc. Neurosci..

[49] Małkowska P. (2024). Positive Effects of Physical Activity on Insulin Signaling. Curr. Issues Mol. Biol..

[50] Sirico F., Bianco A., D’Alicandro G., Castaldo C., Montagnani S., Spera R., Di Meglio F., Nurzynska D. (2018). Effects of Physical Exercise on Adiponectin, Leptin, and Inflammatory Markers in Childhood Obesity: Systematic Review and Meta-Analysis. Child. Obes. Print.

[51] Chiang M.-C., Tsai T.-Y., Wang C.-J. (2023). The Potential Benefits of Quercetin for Brain Health: A Review of Anti-Inflammatory and Neuroprotective Mechanisms. Int. J. Mol. Sci..

[52] Cronin P., Joyce S.A., O’Toole P.W., O’Connor E.M. (2021). Dietary Fibre Modulates the Gut Microbiota. Nutrients.

[53] Guan Z.-W., Yu E.-Z., Feng Q. (2021). Soluble Dietary Fiber, One of the Most Important Nutrients for the Gut Microbiota. Molecules.

[54] Hoscheidt S., Sanderlin A.H., Baker L.D., Jung Y., Lockhart S., Kellar D., Whitlow C.T., Hanson A.J., Friedman S., Register T. (2022). Mediterranean and Western Diet Effects on Alzheimer’s Disease Biomarkers, Cerebral Perfusion, and Cognition in Mid-Life: A Randomized Trial. Alzheimers Dement. J. Alzheimers Assoc..

[55] Kousparou C., Fyrilla M., Stephanou A., Patrikios I. (2023). DHA/EPA (Omega-3) and LA/GLA (Omega-6) as Bioactive Molecules in Neurodegenerative Diseases. Int. J. Mol. Sci..

[56] Sherzai A.Z., Sherzai A.N., Sherzai D. (2023). A Systematic Review of Omega-3 Consumption and Neuroprotective Cognitive Outcomes. Am. J. Lifestyle Med..

[57] Wang Y., Miao F., Wang J., Zheng M., Yu F., Yi Y. (2024). The Ameliorative and Neuroprotective Effects of Dietary Fibre on Hyperuricaemia Mice: A Perspective from Microbiome and Metabolome. Br. J. Nutr..

[58] Winiarska-Mieczan A., Kwiecień M., Jachimowicz-Rogowska K., Donaldson J., Tomaszewska E., Baranowska-Wójcik E. (2023). Anti-Inflammatory, Antioxidant, and Neuroprotective Effects of Polyphenols—Polyphenols as an Element of Diet Therapy in Depressive Disorders. Int. J. Mol. Sci..

[59] Gronowska-Senger A. (2013). Przewodnik Metodyczny Badań Sposobu Żywienia: Praca Zbiorowa.

[60] Szponar L., Wolnicka K., Rychlik E. (2008). Album Fotografii Produktów i Potraw.

[61] Jarosz M., Rychlik E., Stoś K., Charzewska J. (2020). Normy Żywienia Dla Populacji Polski i Ich Zastosowanie.

[62] Laudisio A., Antonelli Incalzi R., Gemma A., Marzetti E., Pozzi G., Padua L., Bernabei R., Zuccalà G. (2018). Definition of a Geriatric Depression Scale Cutoff Based upon Quality of Life: A Population-Based Study. Int. J. Geriatr. Psychiatry.

[63] Norris D., Clark M.S., Shipley S. (2016). The Mental Status Examination. Am. Fam. Physician.

[64] Tafiadis D., Ziavra N., Prentza A., Siafaka V., Zarokanelou V., Voniati L., Konitsiotis S. (2021). The Tuokko Version of the Clock Drawing Test: A Validation Study in the Greek Population. J. Clin. Exp. Neuropsychol..

[65] Sacktor N.C., Wong M., Nakasujja N., Skolasky R.L., Selnes O.A., Musisi S., Robertson K., McArthur J.C., Ronald A., Katabira E. (2005). The International HIV Dementia Scale: A New Rapid Screening Test for HIV Dementia. AIDS Lond. Engl..

[66] Buelt A., Richards A., Jones A.L. (2021). Hypertension: New Guidelines from the International Society of Hypertension. Am. Fam. Physician.

[67] Rychlik E., Stoś K., Woźniak A., Mojskiej H. (2024). Normy Żywienia dla Populacji Polski.

[68] Pandarek Zalecenia Kliniczne Dotyczące Postępowania u osób z Cukrzycą 2024 Stanowisko Polskiego Towarzystwa Diabetologicznego. https://ptdiab.pl/zalecenia-ptd/zalecania-aktywni-czlonkowie-2024.

[69] Solnica B., Sygitowicz G., Sitkiewicz D., Jóźwiak J., Kasperczyk S., Broncel M., Wolska A., Odrowąż-Sypniewska G., Banach M. (2024). 2024 Guidelines of the Polish Society of Laboratory Diagnostics and the Polish Lipid Association on Laboratory Diagnostics of Lipid Metabolism Disorders. Arch. Med. Sci..

[70] Franceschi C., Campisi J. (2014). Chronic Inflammation (Inflammaging) and Its Potential Contribution to Age-Associated Diseases. J. Gerontol. Ser. A.

[71] Mazzonna F., Peracchi F. (2012). Ageing, Cognitive Abilities and Retirement. Eur. Econ. Rev..

[72] Singh A.S., Chin A Paw M.J.M., Bosscher R.J., van Mechelen W. (2006). Cross-Sectional Relationship between Physical Fitness Components and Functional Performance in Older Persons Living in Long-Term Care Facilities. BMC Geriatr..

[73] Singh G.K., Hiatt R.A. (2006). Trends and Disparities in Socioeconomic and Behavioural Characteristics, Life Expectancy, and Cause-Specific Mortality of Native-Born and Foreign-Born Populations in the United States, 1979–2003. Int. J. Epidemiol..

[74] Phillips C., Fahimi A. (2018). Immune and Neuroprotective Effects of Physical Activity on the Brain in Depression. Front. Neurosci..

[75] Barter P., Gotto A.M., LaRosa J.C., Maroni J., Szarek M., Grundy S.M., Kastelein J.J.P., Bittner V., Fruchart J.-C. (2007). HDL Cholesterol, Very Low Levels of LDL Cholesterol, and Cardiovascular Events. N. Engl. J. Med..

[76] Quispe R., Elshazly M.B., Zhao D., Toth P.P., Puri R., Virani S.S., Blumenthal R.S., Martin S.S., Jones S.R., Michos E.D. (2020). TC/HDL-C Ratio Discordance with LDL-C and Non-HDL-C and Incidence of Atherosclerotic Cardiovascular Disease in Primary Prevention: The ARIC Study. Eur. J. Prev. Cardiol..

[77] Bull F.C., Al-Ansari S.S., Biddle S., Borodulin K., Buman M.P., Cardon G., Carty C., Chaput J.-P., Chastin S., Chou R. (2020). World Health Organization 2020 Guidelines on Physical Activity and Sedentary Behaviour. Br. J. Sports Med..

[78] Calabresi L., Franceschini G. (2010). Lecithin:Cholesterol Acyltransferase, High-Density Lipoproteins, and Atheroprotection in Humans. Trends Cardiovasc. Med..

[79] Earnest C.P., Artero E.G., Sui X., Lee D., Church T.S., Blair S.N. (2013). Maximal Estimated Cardiorespiratory Fitness, Cardiometabolic Risk Factors, and Metabolic Syndrome in the Aerobics Center Longitudinal Study. Mayo Clin. Proc..

[80] Harrison M., Moyna N.M., Zderic T.W., O’Gorman D.J., McCaffrey N., Carson B.P., Hamilton M.T. (2012). Lipoprotein Particle Distribution and Skeletal Muscle Lipoprotein Lipase Activity after Acute Exercise. Lipids Health Dis..

[81] Mann S., Beedie C., Jimenez A. (2014). Differential Effects of Aerobic Exercise, Resistance Training and Combined Exercise Modalities on Cholesterol and the Lipid Profile: Review, Synthesis and Recommendations. Sports Med..

[82] Riedl I., Yoshioka M., Nishida Y., Tobina T., Paradis R., Shono N., Tanaka H., St-Amand J. (2010). Regulation of Skeletal Muscle Transcriptome in Elderly Men after 6 Weeks of Endurance Training at Lactate Threshold Intensity. Exp. Gerontol..

[83] da Silva R.C., Diniz M.d.F.H.S., Alvim S., Vidigal P.G., Fedeli L.M.G., Barreto S.M. (2016). Physical Activity and Lipid Profile in the ELSA-Brasil Study. Arq. Bras. Cardiol..

[84] Pelliccia A., Sharma S., Gati S., Bäck M., Börjesson M., Caselli S., Collet J.-P., Corrado D., Drezner J.A., Halle M. (2021). 2020 ESC Guidelines on Sports Cardiology and Exercise in Patients with Cardiovascular Disease: The Task Force on Sports Cardiology and Exercise in Patients with Cardiovascular Disease of the European Society of Cardiology (ESC). Eur. Heart J..

[85] Riddell M.C., Zaharieva D.P., Yavelberg L., Cinar A., Jamnik V.K. (2015). Exercise and the Development of the Artificial Pancreas: One of the More Difficult Series of Hurdles. J. Diabetes Sci. Technol..

[86] Tagougui S., Taleb N., Rabasa-Lhoret R. (2019). The Benefits and Limits of Technological Advances in Glucose Management Around Physical Activity in Patients Type 1 Diabetes. Front. Endocrinol..

[87] Di Liegro C.M., Schiera G., Proia P., Di Liegro I. (2019). Physical Activity and Brain Health. Genes.

[88] Hill T., Polk J.D. (2019). BDNF, Endurance Activity, and Mechanisms Underlying the Evolution of Hominin Brains. Am. J. Phys. Anthropol..

[89] Mattson M.P., Maudsley S., Martin B. (2004). A Neural Signaling Triumvirate That Influences Ageing and Age-Related Disease: Insulin/IGF-1, BDNF and Serotonin. Ageing Res. Rev..

[90] Golden E., Emiliano A., Maudsley S., Windham B.G., Carlson O.D., Egan J.M., Driscoll I., Ferrucci L., Martin B., Mattson M.P. (2010). Circulating Brain-Derived Neurotrophic Factor and Indices of Metabolic and Cardiovascular Health: Data from the Baltimore Longitudinal Study of Aging. PLoS ONE.

[91] Knaepen K., Goekint M., Heyman E.M., Meeusen R. (2010). Neuroplasticity—Exercise-Induced Response of Peripheral Brain-Derived Neurotrophic Factor: A Systematic Review of Experimental Studies in Human Subjects. Sports Med..

[92] Gholami F., Mesrabadi J., Iranpour M., Donyaei A. (2025). Exercise training alters resting brain-derived neurotrophic factor concentration in older adults: A systematic review with meta-analysis of randomized-controlled trials. Exp. Gerontol..

[93] Sakuma K., Yamaguchi A. (2011). The Recent Understanding of the Neurotrophin’s Role in Skeletal Muscle Adaptation. J. Biomed. Biotechnol..

[94] Huang T., Larsen K.T., Ried-Larsen M., Møller N.C., Andersen L.B. (2014). The Effects of Physical Activity and Exercise on Brain-Derived Neurotrophic Factor in Healthy Humans: A Review. Scand. J. Med. Sci. Sports.

[95] Cunha C., Brambilla R., Thomas K.L. (2010). A Simple Role for BDNF in Learning and Memory?. Front. Mol. Neurosci..

[96] Liu P.Z., Nusslock R. (2018). Exercise-Mediated Neurogenesis in the Hippocampus via BDNF. Front. Neurosci..

[97] Chalimoniuk M., Langfort J. (2007). The Effect of Subchronic, Intermittent L-DOPA Treatment on Neuronal Nitric Oxide Synthase and Soluble Guanylyl Cyclase Expression and Activity in the Striatum and Midbrain of Normal and MPTP-Treated Mice. Neurochem. Int..

[98] Cisek-Woźniak A., Mruczyk K., Wójciak R.W. (2021). The Association between Physical Activity and Selected Parameters of Psychological Status and Dementia in Older Women. Int. J. Environ. Res. Public. Health.

[99] Colcombe S., Kramer A.F. (2003). Fitness Effects on the Cognitive Function of Older Adults: A Meta-Analytic Study. Psychol. Sci..

[100] Ekblom M.M., Ekblom Ö.B., Börjesson M., Bergström G., Jern C., Wallin A. (2019). Device-Measured Sedentary Behavior, Physical Activity and Aerobic Fitness Are Independent Correlates of Cognitive Performance in Healthy Middle-Aged Adults—Results from the SCAPIS Pilot Study. Int. J. Environ. Res. Public. Health.

[101] Nakagawa T., Koan I., Chen C., Matsubara T., Hagiwara K., Lei H., Hirotsu M., Yamagata H., Nakagawa S. (2020). Regular Moderate- to Vigorous-Intensity Physical Activity Rather Than Walking Is Associated with Enhanced Cognitive Functions and Mental Health in Young Adults. Int. J. Environ. Res. Public. Health.

[102] Wrann C.D., White J.P., Salogiannnis J., Laznik-Bogoslavski D., Wu J., Ma D., Lin J.D., Greenberg M.E., Spiegelman B.M. (2013). Exercise Induces Hippocampal BDNF through a PGC-1α/FNDC5 Pathway. Cell Metab..

[103] Wang X., Feng S., Deng Q., Wu C., Duan R., Yang L. (2025). The Role of Estrogen in Alzheimer’s Disease Pathogenesis and Therapeutic Potential in Women. Mol. Cell. Biochem..

[104] Ortega R.M., Jiménez Ortega A.I., Martínez García R.M., Cuadrado Soto E., Aparicio A., López-Sobaler A.M. (2021). ^a^; Jiménez Ortega, A.I.; Martínez García, R.M.; Cuadrado Soto, E.; Aparicio, A.; López-Sobaler, A.M. [Nutrition in the prevention and control of osteoporosis]. Nutr. Hosp..

[105] Annevelink C.E., Sapp P.A., Petersen K.S., Shearer G.C., Kris-Etherton P.M. (2023). Diet-Derived and Diet-Related Endogenously Produced Palmitic Acid: Effects on Metabolic Regulation and Cardiovascular Disease Risk. J. Clin. Lipidol..

[106] Ardisson Korat A.V., Shea M.K., Jacques P.F., Sebastiani P., Wang M., Eliassen A.H., Willett W.C., Sun Q. (2024). Dietary Protein Intake in Midlife in Relation to Healthy Aging—Results from the Prospective Nurses’ Health Study Cohort. Am. J. Clin. Nutr..

[107] Nunes E.A., Colenso-Semple L., McKellar S.R., Yau T., Ali M.U., Fitzpatrick-Lewis D., Sherifali D., Gaudichon C., Tomé D., Atherton P.J. (2022). Systematic Review and Meta-Analysis of Protein Intake to Support Muscle Mass and Function in Healthy Adults. J. Cachexia Sarcopenia Muscle.

[108] Traylor D.A., Gorissen S.H.M., Phillips S.M. (2018). Perspective: Protein Requirements and Optimal Intakes in Aging: Are We Ready to Recommend More Than the Recommended Daily Allowance?. Adv. Nutr. Bethesda Md.

[109] Astrup A., Magkos F., Bier D.M., Brenna J.T., de Oliveira Otto M.C., Hill J.O., King J.C., Mente A., Ordovas J.M., Volek J.S. (2020). Saturated Fats and Health: A Reassessment and Proposal for Food-Based Recommendations: JACC State-of-the-Art Review. J. Am. Coll. Cardiol..

[110] Feingold K.R., Feingold K.R., Anawalt B., Blackman M.R., Boyce A., Chrousos G., Corpas E., de Herder W.W., Dhatariya K., Dungan K., Hofland J. (2000). The Effect of Diet on Cardiovascular Disease and Lipid and Lipoprotein Levels. Endotext.

[111] Ozen E., Mihaylova R., Weech M., Kinsella S., Lovegrove J.A., Jackson K.G. (2022). Association between Dietary Saturated Fat with Cardiovascular Disease Risk Markers and Body Composition in Healthy Adults: Findings from the Cross-Sectional BODYCON Study. Nutr. Metab..

[112] Yang L., Yang C., Chu C., Wan M., Xu D., Pan D., Xia H., Wang S.K., Shu G., Chen S. (2022). Beneficial Effects of Monounsaturated Fatty Acid-Rich Blended Oils with an Appropriate Polyunsaturated/Saturated Fatty Acid Ratio and a Low n-6/n-3 Fatty Acid Ratio on the Health of Rats. J. Sci. Food Agric..

[113] Zinöcker M.K., Svendsen K., Dankel S.N. (2021). The Homeoviscous Adaptation to Dietary Lipids (HADL) Model Explains Controversies over Saturated Fat, Cholesterol, and Cardiovascular Disease Risk. Am. J. Clin. Nutr..

[114] Amerkamp J., Benli S., Isenmann E., Brinkmann C. (2024). Optimizing the Lifestyle of Patients with Type 2 Diabetes Mellitus—Systematic Review on the Effects of Combined Diet-and-Exercise Interventions. Nutr. Metab. Cardiovasc. Dis. NMCD.

[115] Hironaka J., Hamaguchi M., Ichikawa T., Nakajima H., Okamura T., Majima S., Senmaru T., Okada H., Ushigome E., Nakanishi N. (2024). Low-Carbohydrate Diets in East Asians with Type 2 Diabetes: A Systematic Review and Meta-Analysis of Randomized Controlled Trials. J. Diabetes Investig..

[116] Skoczek-Rubińska A., Muzsik-Kazimierska A., Chmurzynska A., Jamka M., Walkowiak J., Bajerska J. (2021). Inflammatory Potential of Diet Is Associated with Biomarkers Levels of Inflammation and Cognitive Function among Postmenopausal Women. Nutrients.

[117] Thomsen M.N., Skytte M.J., Samkani A., Carl M.H., Weber P., Astrup A., Chabanova E., Fenger M., Frystyk J., Hartmann B. (2022). Dietary Carbohydrate Restriction Augments Weight Loss-Induced Improvements in Glycaemic Control and Liver Fat in Individuals with Type 2 Diabetes: A Randomised Controlled Trial. Diabetologia.

[118] Gill S.K., Rossi M., Bajka B., Whelan K. (2021). Dietary Fibre in Gastrointestinal Health and Disease. Nat. Rev. Gastroenterol. Hepatol..

[119] Pérez-Jiménez J. (2024). Dietary Fiber: Still Alive. Food Chem..

[120] Antoni R. (2023). Dietary Saturated Fat and Cholesterol: Cracking the Myths around Eggs and Cardiovascular Disease. J. Nutr. Sci..

[121] Jung E., Kong S.Y., Ro Y.S., Ryu H.H., Shin S.D. (2022). Serum Cholesterol Levels and Risk of Cardiovascular Death: A Systematic Review and a Dose-Response Meta-Analysis of Prospective Cohort Studies. Int. J. Environ. Res. Public. Health.

[122] Schade D.S., Shey L., Eaton R.P. (2020). Cholesterol Review: A Metabolically Important Molecule. Endocr. Pract. Off. J. Am. Coll. Endocrinol. Am. Assoc. Clin. Endocrinol..

[123] Soliman G.A. (2018). Dietary Cholesterol and the Lack of Evidence in Cardiovascular Disease. Nutrients.

